# Are there roles for heterogeneous ribosomes during sleep in the rodent brain?

**DOI:** 10.3389/fmolb.2022.1008921

**Published:** 2022-10-06

**Authors:** Isla M. Buchanan, Trevor M. Smith, André P. Gerber, Julie Seibt

**Affiliations:** ^1^ Integrated Master Programme in Biochemistry, University of Surrey, Guildford, United Kingdom; ^2^ Department of Microbial Sciences, School of Biosciences and Medicine, Faculty of Health and Medical Sciences, University of Surrey, Guildford, United Kingdom; ^3^ Surrey Sleep Research Centre, University of Surrey, Guildford, United Kingdom

**Keywords:** ribosomal protein, ribosome heterogeneity, neuron, neurites, sleep, brain plasticity, synapse

## Abstract

The regulation of mRNA translation plays an essential role in neurons, contributing to important brain functions, such as brain plasticity and memory formation. Translation is conducted by ribosomes, which at their core consist of ribosomal proteins (RPs) and ribosomal RNAs. While translation can be regulated at diverse levels through global or mRNA-specific means, recent evidence suggests that ribosomes with distinct configurations are involved in the translation of different subsets of mRNAs. However, whether and how such proclaimed ribosome heterogeneity could be connected to neuronal functions remains largely unresolved. Here, we postulate that the existence of heterologous ribosomes within neurons, especially at discrete synapses, subserve brain plasticity. This hypothesis is supported by recent studies in rodents showing that heterogeneous RP expression occurs in dendrites, the compartment of neurons where synapses are made. We further propose that sleep, which is fundamental for brain plasticity and memory formation, has a particular role in the formation of heterologous ribosomes, specialised in the translation of mRNAs specific for synaptic plasticity. This aspect of our hypothesis is supported by recent studies showing increased translation and changes in RP expression during sleep after learning. Thus, certain RPs are regulated by sleep, and could support different sleep functions, in particular brain plasticity. Future experiments investigating cell-specific heterogeneity in RPs across the sleep-wake cycle and in response to different behaviour would help address this question.

## Local translation in neurons: evidence and functions

**FIGURE 1:**
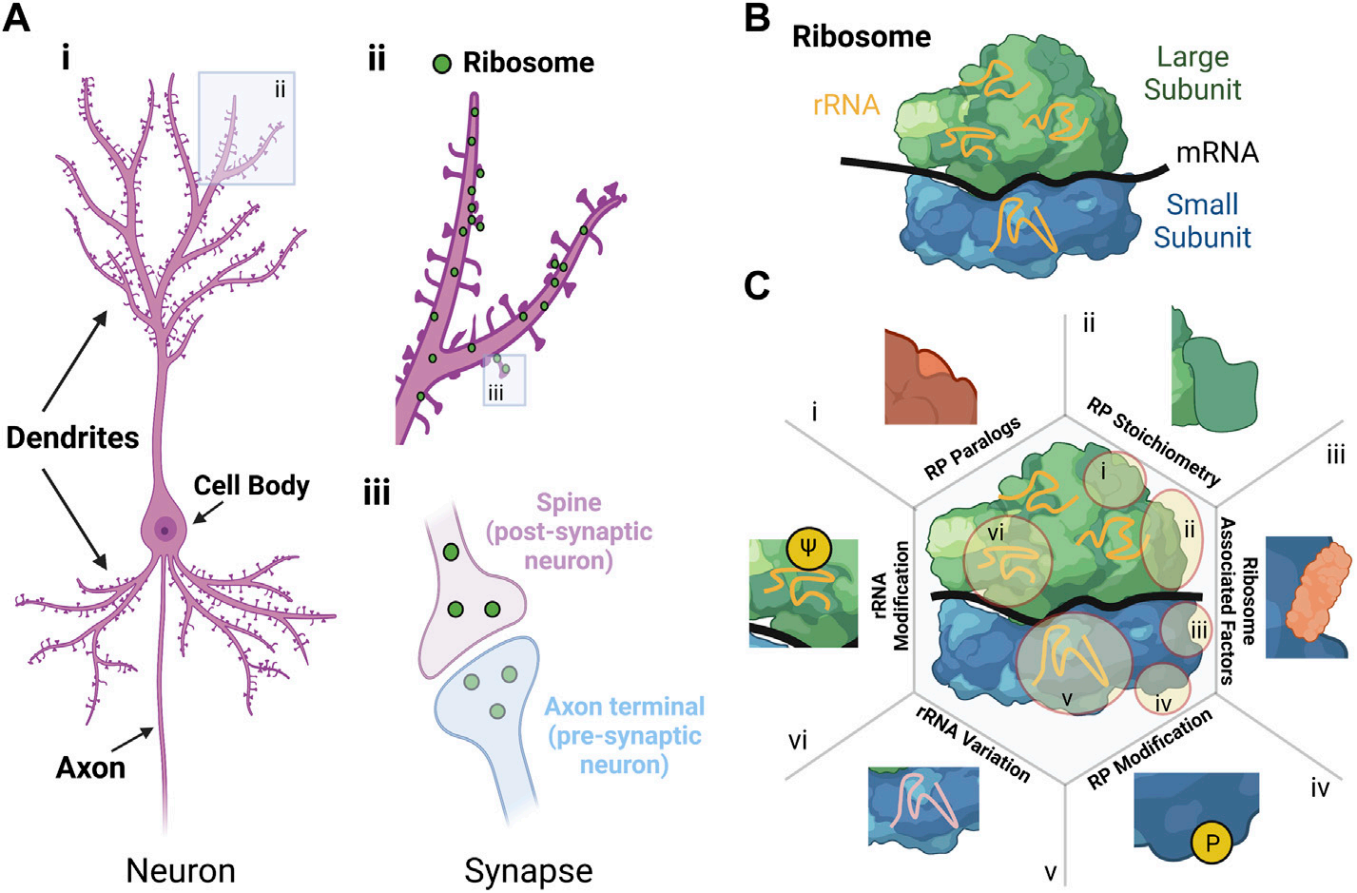
Sources and effect of ribosomal heterogeneity in neurons. **(A)** Morphology of a neuron. i) Schematic of a neuron showing the cell body, dendrites and axon. ii) Close-up schematic of dendrites with ribosomes shown as green dots located both in and adjacent to synapses. iii) Close-up schematic of the synapse with ribosomes shown as green dots in the cytoplasm in both the post-synaptic neuron (i.e., spine in purple) and pre-synaptic neuron (i.e., axon terminal in blue). **(B)** Structure of the ribosome with the large subunit (green), small subunit (blue), ribosomal RNA (rRNA in orange), and messenger RNA (mRNA in black). For the large and small subunit, the different colours indicate different ribosomal proteins. **(C)** Schematic showing six sources of ribosomal variation, with illustrative examples of how these changes manifest: i) RP paralogs, ii) RP stoichiometry, iii) Ribosomal associated factors, iv) RP modification, v) rRNA variation, and vi) rRNA modification (adapted from (Norris et al., 2021)). Created with BioRender.com.

Neurons are the primary cell type in the nervous system (the other being glia, which provide structural and metabolic support), responsible for transmission of electrochemical signals in the form of action potentials (APs). On average, the volume of a neuron is more than 10,000 times greater than most mammalian cells. Neurons have a complex and polarised morphology with processes (i.e., neurites) which are divided into different compartments called dendrites and axons, that can extend millimetres to meters away from the cell body containing the nucleus. Axons transmit APs to other neurons and dendrites receive APs and release neurotransmitters onto little protrusions called spines (Donato et al., 2019) (Figure 1A). The site of connection between axon termini and dendritic spines is called the synapse (Figure 1A). One neuron can host up to hundred thousand spines on its dendrites and thus receive thousands of contacts from different axons, maximising the number of possible synapses between neurons (Chidambaram et al., 2019; Nishiyama, 2019; Megías et al., 2001; Spruston, 2008).

In neurons, timely production of proteins in individual neurites and synapses is critical for spatial control of cellular function (Perez et al., 2021b). Indeed, using high resolution imaging of neurons *in vivo* and *in vitro*, it has been shown that most pre-synaptic axon termini and post-synaptic spines contain ribosomes, translation factors, and mRNAs (Cajigas et al., 2012; Kitamura et al., 2015; Holt et al., 2019; Sun et al., 2021) (Figure 1A). Local protein synthesis in neurons is required for growth and remodelling of synapses (i.e., synaptic plasticity) (Sutton and Schuman, 2006), which enables critical processes such as brain development, brain plasticity, learning and memory (Banko and Klann, 2008) and recovery from brain injury by promoting synapse-specific production of functionally distinct groups of proteins.

For example, strengthening and weakening of individual synaptic contacts relies on the synthesis of different pools of proteins which ultimately increase or decrease efficiency of communication (Zukin et al., 2009). Local translation of proteins critical for long-term increases in synaptic transmission includes α-amino-3-hydroxy-5-methyl-4-isoxazolepropionic acid (AMPA) receptors which increases the sensitivity of dendrites to incoming stimuli and strengthens individual synapses (Perez et al., 2021b). Similarly, local translation of proteins with key roles in synaptic structure, such as Arc, actin, PSD95 and α/βCaMKII, participates in the structural modification of dendritic spines during plasticity processes (Nakahata and Yasuda, 2018; Newpher et al., 2018).

As modulation of synaptic communication is naturally linked to memory formation, translation regulation is also important for memory (Sutton and Schuman, 2006; Banko and Klann, 2008), as shown in *Drosophila* (Ashraf et al., 2006) and rodents. Inhibition of translation initiation (i.e., mTOR signalling) in the rodent hippocampus, a brain structure important for memory formation, impairs performance in a widely used single trial inhibitory avoidance memory task in rodents (Bekinschtein et al., 2007). While *in vivo* manipulation of local translation at specific synapses remains challenging, experimental and computational studies support the specific involvement of molecular changes in dendrites (Kastellakis and Poirazi, 2019) and spines (Hayashi-Takagi et al., 2015) for memory formation. Altogether, localised mRNA translation within neurons plays a critical role in many brain functions involving the growth and remodelling of synapses. However, a complete picture of the forms and functions of various translation regulatory mechanisms contributing to brain function in neurons remains largely unexplored.

## Translation regulation through heterogeneous ribosomes

The eukaryotic ribosome is a large ribonucleoprotein complex that consists of a large (60S) and a small (40S) subunit that assemble during the initiation step of translation to form complete 80S ribosomes (Figure 1B). The two subunits share four ribosomal RNAs (rRNAs) (Ford, 2020) and 80 ribosomal proteins RPs, 33 of them allocated to the small (RPS) and 47 to the large (RPL) subunit (De La Cruz et al., 2015). Translation can be regulated at diverse levels (e.g., initiation, elongation, or termination) but major impact is given at the initiation step. Thereby, translation can be controlled at a global level or for specific mRNAs, for example through modification of translation initiation factors and specific RNA binding proteins (RBPs) or microRNAs, respectively (Sonenberg and Hinnebusch, 2009; King and Gerber, 2016).

Beside the regulation of translation mediated through those accessory factors, the view that ribosomes are all identical prevailed for decades. However, increasing evidence over the last decade suggests that cell-specific heterogeneous populations of ribosomes could exist and may result in different preferences of individual ribosomes (or “specialised ribosomes”) for the translation of diverse subsets of mRNAs or in the modulation of translation elongation rates (Genuth and Barna, 2018; Gay et al., 2022). Such heterogenous populations of ribosomes can be formed by (Figure 1C **,** (Norris et al., 2021)) 1) the exchange or substitution of RP paralogues (e.g., RPL39 and RPL39L) (Komili et al., 2007), 2) altered RP stoichiometry based on differences in RP expression (Emmott et al., 2019); 3) different ribosome-associated proteins (Simsek et al., 2017), 4) post-translational modifications of RPs (Carroll et al., 2008); 5) rRNA composition through variation of rRNA gene sequences (Locati et al., 2017); and 6) rRNA modifications, such as ribose-methylation or pseudouridylation (ψ) (Natchiar et al., 2017). Consequently, these factors could lead to a cell-specific array of ribosome variants, some of them having specialised functions in translation (Li and Wang, 2020; Gay et al., 2022).

A well-described example of ribosome specialisation concerns RPL38 (Xue et al., 2015). Ribosomes containing RPL38 are required for translation of certain *Homeobox (Hox)* mRNAs that code for proteins defining the body axis and structures (Kondrashov et al., 2011). In developing mice embryos, *Rpl38* transcripts were enriched in certain regions including the face, eye, neural tube (brain and spinal cord precursor) and importantly somites, which are precursors of the axial skeleton. These locations tended to overlap with regions where tissue patterning defects were observed in mice with the “Tail short” (Ts) mutation (which display a short and curled tail, an anteroposterior skeletal patterning defect and several other skeletal abnormalities), a phenotype thought to be caused by *Rpl38* gene mutation (Kondrashov et al., 2011). RPL38 has been reported to control cap-independent translation of *Hox* mRNAs *via* specific internal ribosome entry sites (IRES) (Xue et al., 2015), although results from a recent study suggest that transcriptional promoters or splice sites may instead be responsible for the putative IRES activity in *Hox* genes (Akirtava et al., 2022). However, selective translation through IRES specificity is likely replicated with other RPs (Hertz et al., 2013; Shi et al., 2017; Kampen et al., 2019). Selective capture of ribosomes containing two specific RPs, RPS25, and RPL10A, combined with ribosome profiling, showed that those RPs preferentially translated unique sets of transcripts (Shi et al., 2017). Specifically, RPS25-containing ribosomes were preferentially associated with transcripts coding for proteins involved in the cell cycle and vitamin B12 pathway, while RPL10A-enriched transcripts were associated with extracellular matrix organisation, system development and steroid metabolism (Shi et al., 2017). In the same way that RPL38 is thought to be required for IRES-dependent translation of *Hox* mRNAs (Xue et al., 2015), Shi et al. (2017) found that RPL10A is required for the translation of three mRNAs known to contain IRES elements (*Igf2, App,* and *Chmp2a*). IRES-dependent translation of these transcripts was significantly reduced upon knockdown of *Rpl10a*, but not the control *Rpl29*, indicating that translation of *Igf2*, *App* and *Chmp2a* is attributed to RPL10A.

Further support for the model of ribosome specialisation is provided by examples of human diseases such as ribosomopathies and fragile-X syndrome. Ribosomopathies are pathologies which result from mutations in certain RP genes (Orgebin et al., 2020), with as many as 20 RP genes, including *RPS19,* involved in Diamond-Blackfan anaemia (DBA) (Leger-Silvestre et al., 2005; Da Costa et al., 2020). Besides a deficiency of erythroblasts, about 50% of DBA patients experience other congenital anomalies such as growth retardation, cardiac and urogenital abnormalities, increased risk of cancer, cephalic malformations and learning difficulties (Da Costa et al., 2020; Panda et al., 2020). Fragile-X syndrome is another well-characterised neurodevelopmental disorder associated with intellectual disability and learning difficulties. Excessive translation due to the loss of the RBP fragile-X mental retardation protein (FMRP) caused by CGG triplet repeat expansions in the FMR1 gene is a key mechanism in the disease (Richter et al., 2015). In this regard, a recent study suggests that excessive translation of RPs in neurons reduces the translation of longer-length transcripts coding for proteins contributing to synaptic stability (Seo et al., 2022), supporting the established link between the loss of FMRP and aberrant synaptic plasticity (Sidorov et al., 2013).

While research is needed to determine the biological functions of ribosome heterogeneity, some studies have highlighted its importance in specific cellular processes, in particular during development (Li and Wang, 2020; Norris et al., 2021). Whether ribosome heterogeneity exists and has functions in the brain remains under-investigated.

## Ribosome heterogeneity in neurons

Although specific RPs (e.g., RPL38, RPS25, and RPL10A) have functional roles in different cell types (see above) (Li and Wang, 2020), still very little is known about whether RPs are differentially regulated or expressed in different areas of the brain or in specific brain cells. For example, RP heterogeneity between brain regions has been reported in normal and brain cancer samples (Panda et al., 2020). Conversely, ageing does apparently not drastically influence RP stoichiometry in the cortex, cerebellum and hippocampus when assessed in a mixed cell preparation (Amirbeigarab et al., 2019); this does not, however, exclude the possibility for heterogeneity associated with specific cell types forming those tissues, including neurons and glial cells. In the following sections, we further focus our considerations on ribosome heterogeneity and functions in neurons, which are the brain cells responsible for activity in the brain and are central for information processing supporting cognitive functions.

**FIGURE 2:**
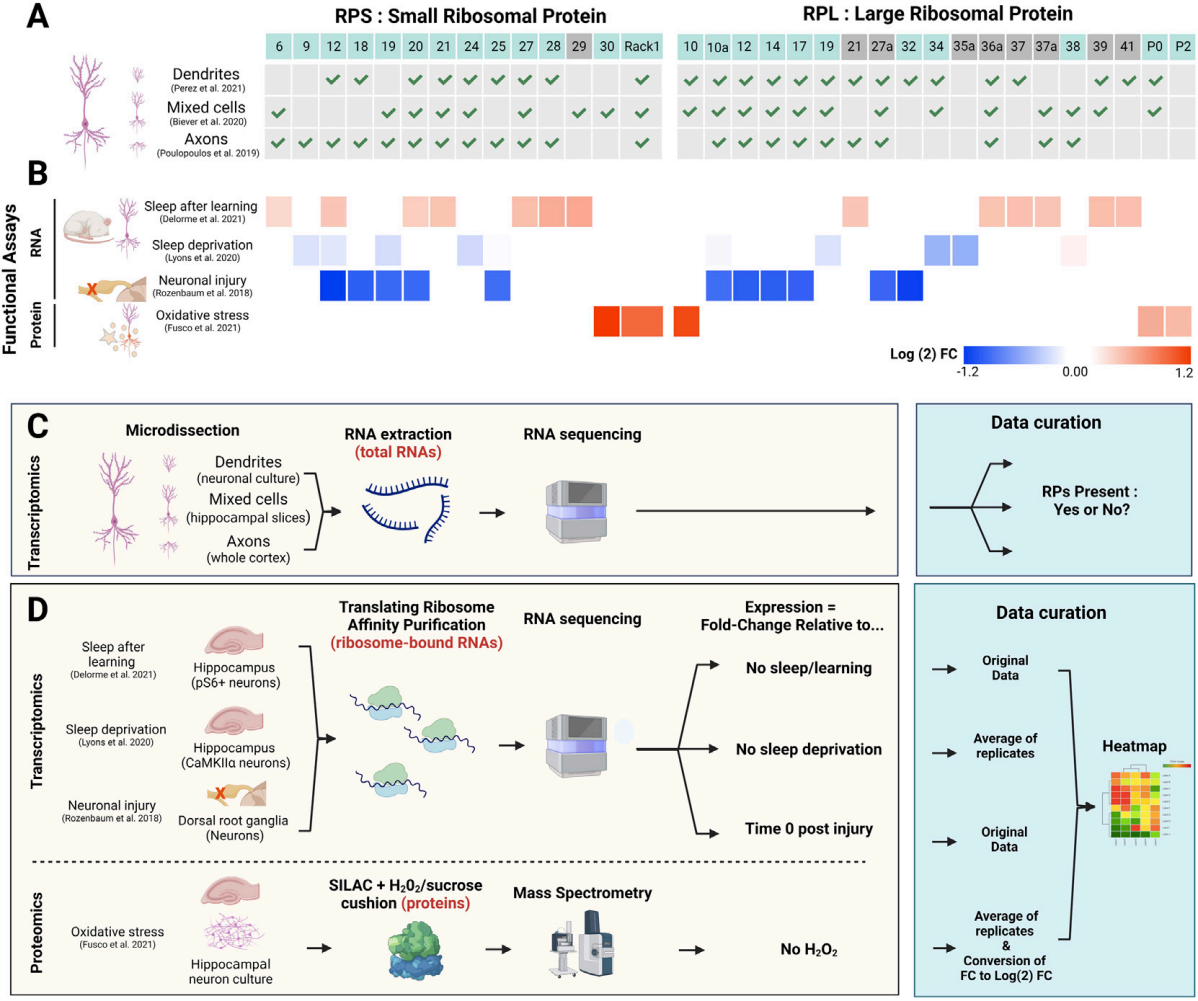
Contribution of RPs to ribosome heterogeneity in neurons and functional significance. **(A)** Table indicating the presence (green check mark) or absence of select RP (top line) mRNAs in whole neurons vs. dendrites or axons, according to previous studies (Poulopoulos et al., 2019; Biever et al., 2020; Perez et al., 2021a). RP location (non-surface: grey boxes; surface: blue boxes) was determined using human ribosome structure (PDB: 4V6X). **(B)** Heatmap (Morpheus, https://software.broadinstitute.org/morpheus) representing heterogeneous expression of selected RPs in neurons from *in vivo* studies in rodents or *in vitro* studies. The colour bar depicts log (2) fold changes (FC) of the particular conditions (indicated on the left) against control condition values (see D and (Rozenbaum et al., 2018; Lyons et al., 2020; Delorme et al., 2021; Fusco et al., 2021) for experimental details). From top to bottom: 1) 13 RP transcripts on membrane-bound ribosomes in activated (pS6+) neurons in the hippocampus following contextual fear conditioning and subsequent sleep in mice (Delorme et al., 2021). 2) 7 RP mRNAs affected by acute (5 h) sleep deprivation in excitatory neurons (CamKIIα+) in the mouse hippocampus (Lyons et al., 2020). 3) The five largest changes for small and large ribosomal subunits (10 RPs) in dorsal root ganglion (DRG) neurons following sciatic nerve injury, 4 h after injury (Rozenbaum et al., 2018). 4) 5 RPs that are significantly upregulated after 0.1 mM H_2_O_2_ treatment of primary neuronal culture; values are average of three biological replicates (Fusco et al., 2021). Additionally, log (2)FC values for RPs previously mentioned in the text and associated with ribosome specialisation (RPS19, RPS25, RPL10A and RPL38) were included for each study if they were present in the original data. **(C,D)** Schematic experimental approaches of the studies performed in neuronal compartments **(C)** relates to **(A)** and functional assays **(D)** relates to **(B)**. The blue boxes specify the type of analysis performed for each study for data visualisation. Created with BioRender.com.

The recent implementation of various transcriptomics and proteomics approaches applied *in vitro* and *in vivo* (Figure 2C) revealed that neurons show differential expression of RPs within the dendritic and axonal sub-compartment compared to a mixed cell population (Poulopoulos et al., 2019; Biever et al., 2020; Perez et al., 2021a) (Figure 2A) and suggest a compartment-specific heterogeneity in RP expression within neurons (Figure 2B). Additionally, an influential study into ribosome specialisation was recently carried out in *Xenopus laevis* showing that remodelling of ribosomes occurred in axons by exchange of locally synthesised RPs (Shigeoka et al., 2019). While ribosome biogenesis typically occurs in the nucleus, this study provides further evidence that ribosomes are dynamic structures.

Several studies have been using the translating ribosome affinity purification (TRAP) method to study neuron-specific changes in the translatome (i.e., pool of translated mRNA). Those studies have provided evidence for differential expression of ribosomal components and RPs in neurons, in particular upon changing conditions/neuronal activation (Figures 2B,D). For instance, more than 1,600 differentially expressed transcripts were identified in dorsal root ganglion (DRG) neurons following sciatic nerve injury (Rozenbaum et al., 2018). Importantly, in lumbar DRG neurons, mRNAs for several RPs were significantly decreased 4 h after injury, with the greatest decrease in expression seen for RPS12 and RPL32 (Figure 2B), and recovery of RP expression after 12 h. Another study in rats utilised single-molecule fluorescence *in situ* hybridisation to show variable expression of 29 different RP mRNAs in dendrites in both hippocampal slices and primary neuronal culture (Fusco et al., 2021). Using an elegant approach combining heavy amino acid labelling (dynamic SILAC) followed by mass spectrometry to label and identify newly synthesised RPs in translating ribosomes, the authors revealed that a subset of 12 RPs showed a dynamic profile of association/dissociation with ribosomes in dendrites, including RPL38 described above. The majority of those 12 RPs are located at the surface of the ribosome, making those proteins susceptible to exchange or post-translational modifications (PTMs) that could—in principle—modulate the function/activity of ribosomes. In fact, many of the RPs showing compartment-specific differences and functional changes are located on the surface of the ribosome (Figure 2). An example of such PTMs of RPs is the phosphorylation of RPS6 in response to a variety of neuronal stimuli, adding the possibility for introduction of compartment specific and activity specific PTM of RPs, propagating functional impact for translation of mRNA subsets (Knight et al., 2012). Finally, changes in the environment (i.e., oxidative stress induced by hydrogen peroxide exposure) elicit five of those 12 RPs to increase their association with the ribosome (Fusco et al., 2021) (Figure 2B), suggesting that physiological stress modifies ribosome composition and function, which could directly affect translation of specific mRNAs. Therefore, other changes in physiological states could also have a significant impact on RP ribosomal protein heterogeneity in neurons.

Altogether, we propose that the existence of heterologous ribosomes within neurons, especially at discrete synapses, may contribute to specific functions, including synaptic plasticity. Dynamic incorporation of RPs to alter ribosome stoichiometry could facilitate rapid formation of specialised ribosomes and enable the translation of subsets of mRNAs involved in the remodelling of synapses. We further postulate that sleep, which is accompanied by major physiological changes in the brain and is an important regulator of the synaptic proteome (Noya et al., 2019), translation and brain plasticity (Seibt and Frank, 2019), could use changes in ribosome heterogeneity and specialised ribosomes to regulate the translation of mRNAs important for synaptic remodelling.

## Sleep, plasticity, and ribosomal proteins

The role of sleep in brain plasticity and memory is well established; mounting evidence shows that sleep enhances the physiological and behavioural changes associated with new experiences (Abel et al., 2013; Rasch and Born, 2013; Raven et al., 2018; Puentes-Mestril et al., 2019). For example, performance in various types of memory tasks in humans and animals is significantly increased when sleep occurs right after learning (Alger et al., 2015; Schmid et al., 2020). Similarly, new sensory experience changes perception in a sleep-dependent manner during development (Frank et al., 2001) and adulthood (Durkin and Aton, 2019). Furthermore, work in the last decade, using high-resolution imaging techniques, has provided strong evidence that sleep influences changes in dendritic spine structure, linked to brain plasticity and memory (Yang et al., 2014; Li et al., 2017; Seibt et al., 2017; Zhou et al., 2020; Aime et al., 2022). Finally, the establishment of our basic sensorimotor system in the central nervous system is thought to largely depend on sleep during early development (Blumberg, 2015). Since long-term changes associated with synaptic plasticity and memory require protein synthesis to persist over time (Davis and Squire, 1984; Costa-Mattioli et al., 2009), sleep may support brain plasticity *via* translation regulation, including specialised ribosomes, for translation of particular subsets of mRNAs.

The current view suggests that experience-dependent transcription (e.g., immediate early genes) (Yap and Greenberg, 2018) occurs preferentially during wakefulness in the nucleus, while mRNA translation occurs mostly during sleep in a distributed manner across neurites (Seibt and Frank, 2019). This is supported by evidence showing that translation rates are increased during sleep in the brain in various species (Ramm and Smith, 1990; Nakanishi et al., 1997) and the expression of regulators of translation initiation and elongation occurs preferentially during sleep (Cirelli et al., 2004; Mackiewicz et al., 2007; Seibt et al., 2012). Moreover, sleep deprivation leads to a decrease in translation initiation with associated memory deficits (Tudor et al., 2016) and pharmacological disruption of translation initiation during sleep impairs experience-dependent synaptic plasticity *in vivo* (Seibt et al., 2012), further suggesting the importance of translation during sleep for brain plasticity and memory consolidation. The underlying pathways for translational control during sleep are still not well-characterised, but global control of translation initiation, *via* the mTORC-1 signalling pathway, seems to be specifically activated during sleep (Seibt et al., 2012; Tudor et al., 2016). (Gingras et al., 1999; Seibt et al., 2012; Tudor et al., 2016) Other factors such as RBPs, microRNAs or non-coding RNAs (ncRNAs) are all potential mechanisms involved in translational control of specific mRNAs during sleep. There is some evidence that sleep deprivation differentially impacts the expression of groups of microRNAs in different parts of the brain, with a trend toward decreased expression in the cortex and increased expression in the hippocampus (Davis et al., 2007). Although this supports a region-specific regulation of translation by the sleep-wake cycle *via* microRNAs, the data on this remain isolated.

Besides global and specific control through signalling pathways and RBPs/ncRNAs, respectively, whether the formation of specialised ribosomes could also contribute to selected translation during sleep remains unclear. However, sleep-dependent translational changes were examined within neurons in the hippocampus after learning in mice (Delorme et al., 2021) (Figure 2C). Using TRAP, ribosome-associated transcripts were identified from different subcellular fractions of neurons (i.e., cytosolic vs. membrane-associated ribosomes). Sleep deprivation primarily affected mRNA translated in the cytosol, while learning mainly altered transcripts on membrane-bound ribosomes, suggesting a first level of translational specificity within neurons. Importantly, sleep after learning showed increased translation of membrane-bound transcripts, including mRNAs of 13 RPs, such as *Rps27* and *Rps28,* with ∼50% of the RPs located at the ribosome surface (Figure 2B) (Delorme et al., 2021). These changes were specifically allocated to sleep as RP mRNA expression did not increase if sleep was prevented after learning. Differential RP translation during sleep may thus support compartmentalised heterogenous populations of ribosomes occurring through exchange and incorporation of RPs at the surface of ribosomes. Another, indirect, evidence supporting a role for sleep in increased translation of RPs was provided by a study investigating the impact of sleep deprivation (SD) on hippocampal neurons using TRAP in mice (Lyons et al., 2020). Following 5 h of SD, 198 mRNAs showed differential association with ribosomes compared to sleep control mice (Lyons et al., 2020). Certain RPs showed a trend toward decreased expression; transcripts for RPs previously associated with ribosome specialisation such as *Rps25*, *Rpl10a* and *Rpl38* displayed little to no change, but other RP mRNAs like *Rpl34* and *Rpl35a* were more affected (Figure 2B). Although none of these changes were found to be significant, the data nevertheless suggests that sleep may promote the expression of particular RPs as short sleep deprivation tends to reduce their translation (Lyons et al., 2020).

Although the data are still sparse, they do support a specific effect of sleep on differential RP expression in brain regions important for memory. The variability of changes observed in the various physiological and behavioural paradigms align with the idea that different neuronal functions are accompanied by expression of different ribosomes, which may favour formation of specialised ribosomes.

## Discussion and conclusions

Several studies revealed differences in RP expression in neurons, some of them specifically during sleep. Furthermore, many RPs are located on the ribosomal surface, adding the possibility for alternative integration or exchange with other RPs. Changes in RP expression and stoichiometry could contribute to the remodelling of neuronal networks and other processes that benefit from sleep, such as general metabolism and membrane repair (Mackiewicz et al., 2007; Anafi et al., 2019), energy conservation (Roth et al., 2010), mood and stress restoration (Goldstein and Walker, 2014), or the clearance of toxins (Xie et al., 2013; van Alphen et al., 2021). While the incorporation and presence of specialised ribosomes in neurons needs to be shown, it may allow for translation of different subsets of mRNAs across individual neuronal compartments and sleep stages.

Experience-dependent plasticity, including memory, leads to the formation or strengthening of certain synapses, whereas others are weakened or even removed. (Yang et al., 2009; Sanders et al., 2012; El-Boustani et al., 2018). Consequently, within the same dendrites, some spines grow while others retract. These dynamic processes involve different mechanisms and proteins, which are - at least in part - instructed by the synthesis of process-relevant proteins. Furthermore, sleep is composed of two different stages, rapid-eye movement (REM) and non-REM sleep, which alternate within minutes in rodents (i.e., one NREM-REM cycle ∼5–10 min) (Trachsel et al., 1991). Interestingly, those phases are coupled with the formation and removal of synapses (Bellesi and De Vivo, 2020; Sun et al., 2020), occurring preferentially during NREM and REM sleep phases, respectively (Yang et al., 2014; Zhou et al., 2020). Hence, assembly of specialised ribosomes during NREM and/or REM sleep could be well-suited to quickly adjust mRNA translation, thereby promoting and/or consolidating the bi-directional plasticity at synapses. How specialised ribosomes are established, controlled, and could become selectively activated during particular sleep phases remains to be uncovered and may be linked to specific brain waves during NREM and REM sleep, known to reactivate selected circuits and enhance memory (Poe, 2017; Ngo et al., 2020; Zhou et al., 2020; Skelin et al., 2021).

The currently available data relate mostly to differential mRNA expression of RPs (Figure 2). However, the data does not necessarily show that the RPs are also synthesised and incorporated into active ribosomes, possibly contributing to ribosome heterogeneity. Thus, besides establishing and monitoring specific RP synthesis in neurons, several questions need to be addressed in the future: 1) Does RP heterogeneity take place in neuronal sub-compartments? 2) How does RP heterogeneity in specific neuronal compartments impact the translation of subsets of mRNAs? 3) How do brain states modulate ribosome heterogeneity to generate specialised ribosomes? Finally, besides neurons there are other important brain cells, such as glia (e.g., astrocytes, microglia) and the vasculature, where ribosome heterogeneity may apply and entail specialised functions (Knight et al., 2012; Bellesi et al., 2015).

In the future, we expect that fundamental questions will be addressed *in vitro* using either brain slices or primary neuronal cultures. For instance, the application of proximity-based labelling techniques could allow the isolation of proteins/RNAs in the vicinity of a target molecule (e.g., RPs and mRNAs) (Padron et al., 2019; Ramanathan et al., 2019), which should identify the spatial partners present under different physiological conditions to help understand function. Furthermore, we have shown evidence of translating RPs in different physiological states (Figure 2). Combining puromycylation (Tom Dieck et al., 2015), to tag newly synthesised proteins, with specific antibodies (i.e. RPs) could help understand the spatial location of these newly translated RPs, to further understand RNA/protein interactions (Weissinger et al., 2021) in different brain cells and neuronal compartments *in vitro*. Obtaining functional *in vivo* data remains the gold standard for understanding molecular mechanisms linked to behaviour, including sleep. However, due to technical challenges of *in vivo* pharmacology (e.g., diffusion, dilution, biochemical reactions), the application of the above-mentioned methods remains difficult (Uezu et al., 2016) and further advances for the relevant techniques are required. For example, *in vivo* imaging of small RPs and RNA (Tatavarty et al., 2012; Tutucci et al., 2018; Cawte et al., 2020) is currently difficult, but not impossible (Grimm et al., 2017; Wegner et al., 2017; Hrabetova et al., 2018). Technical improvements such as brighter and more photostable fluorophores, higher resolution imaging, and better access to the tissue of interest (Tutucci et al., 2018; Das et al., 2021) may be key developments to advance the field, allowing better monitoring of RP localisation in cells. Furthermore, improved methods for genetic manipulations (Raguram et al., 2022), to target RPs with fewer off-target side effects, may allow tagging of different ribosomal components at the same time, facilitating isolation of different ribosome complexes from the same cell with TRAP. At the end, those approaches could be combined with behavioural and sleep manipulations, opening the paths towards fundamental understanding of the functional impact of ribosome heterogeneity in complex physiological processes.

## Data Availability

The original contributions presented in the study are included in the article; further enquiries can be directed to the corresponding authors.
